# Genomic Insights and Antimicrobial Resistance Profiling of Diarrheagenic *Escherichia coli* in Bhutan: A Retrospective Whole-Genome Sequencing Study

**DOI:** 10.3390/microorganisms14071541

**Published:** 2026-07-15

**Authors:** Tshering Dorji, Kunzang Dorji, Kinley Gyem, Sonam Gyeltshen, Yoshio Yamaoka, Takashi Matsumoto

**Affiliations:** 1 Royal Centre for Disease Control, Ministry of Health, Royal Government of Bhutan, Serbithang, Thimphu 11001, Bhutan; cerorziks@gmail.com (T.D.); kinley09@gmail.com (K.G.); sgyeltshen1994@gmail.com (S.G.); 2 Department of Environmental and Preventive Medicine, Faculty of Medicine, Oita University, Yufu 879-5593, Japan; yyamaoka@oita-u.ac.jp; 3 The Research Center for Global and Local Infectious Diseases (RCGLID), Oita University, Yufu 879-5593, Japan

**Keywords:** AMR, diarrhea, sequencing, MLST, Bhutan

## Abstract

Diarrheal disease remains a significant public health concern in Bhutan; however, the genomic epidemiology of the circulating diarrhoeagenic *Escherichia coli* (DEC) strain remains poorly understood. This study characterized the genomic diversity, antimicrobial resistance (AMR), and virulence determinants of DEC isolates using whole-genome sequencing (WGS). DEC isolates recovered from stool samples and collected through Bhutan’s National Diarrheal Disease Surveillance sentinel hospitals during 2023 were identified by a multiplex polymerase chain reaction, tested for antimicrobial susceptibility using the Kirby–Bauer disc diffusion method, and sequenced on the Illumina MiSeq platform. Genomes were analyzed using the Bohra pipeline to determine pathotypes, phylogeny, multilocus sequence types, serotypes, virulence factors, and AMR genes. Of the 29 DEC isolates, 27 were confirmed by WGS and enteropathogenic *E. coli* (37.0%) and enteroaggregative *E. coli* (33.3%) were the predominant pathotypes. Isolates exhibited extensive genetic diversity, representing phylogroups A and B1 and 22 serotypes. Phenotypic resistance to β-lactams was common, with 25.9% of isolates carrying *bla*_CTX-M-15_. Virulence profiling identified diverse adhesins, toxins, iron acquisition systems, and type III secretion system components. DEC isolates in Bhutan comprise a genetically diverse population with a concerning convergence of virulence determinants and multidrug resistance. The findings underscore the strengthening of sustained genomic surveillance to monitor AMR and genomic epidemiology of bacterial pathogens.

## 1. Introduction

Diarrheal diseases are a leading cause of morbidity and mortality in children under five years of age in developing countries, with an estimated 500,000 deaths annually [1,2]. Globally, particularly in South Asia and Africa, it remains a major public health challenge despite significant improvements in sanitation and healthcare infrastructure, a situation further worsened by the rapid emergence and spread of antimicrobial resistance [3]. In Bhutan, diarrhea has consistently ranked among the top ten reported diseases over the past five years, with 33,505 cases reported in 2023 alone, and is identified as the second most frequently notified condition nationally [4,5]. Analysis of diarrheal cases from 2016 to 2022 showed that over 90% of stool samples were positive for at least one enteric pathogen, with diarrhoeagenic *Escherichia coli* (DEC) identified as the most common bacterial cause (8.4%). Substantial antimicrobial resistance was observed among bacterial isolates, including high resistance to amoxicillin (80.3%) and ampicillin (77.4%), with many strains exhibiting multidrug resistance [5,6].

Globally, DEC is recognized as one of the most common bacterial causes of diarrhea in the Global Enteric Multicentre Study (GEMS) [7]. *Escherichai coli* (*E. coli*) is a Gram-negative, rod-shaped bacterium from the family Enterobacteriaceae, typically a harmless commensal organism residing in the gastrointestinal tract of warm-blooded animals. However, certain lineages carry specific virulence factors (VFs) that result in these lineages being recognized as highly diverse and adaptable pathogens. These VFs are often encoded on mobile genetic elements, facilitating the horizontal movement of these determinants and contributing to lineage diversity [8]. DEC can cause gastrointestinal symptoms including watery diarrhoea, nausea, vomiting and abdominal cramps, and is generally classified into seven different pathotypes, which include enteropathogenic *E. coli* (EPEC), Enterohemorrhagic *E. coli* (EHEC), enterotoxigenic *E. coli* (ETEC), enteroinvasive *E. coli* (EIEC), enteroaggregative *E. coli* (EAEC), diffusely adherent *E. coli* (DAEC) and Adherent-Invasive *E. coli* (AIEC) [8,9]. Antimicrobial susceptibility testing is essential for selecting appropriate antibiotics for patients; however, the phenotypic methods are cumbersome and time-consuming. Consequently, polymerase chain reaction (PCR)-based rapid typing of antimicrobial resistance (AMR) genes has been developed and implemented [10]. However, given the complex drug resistance mechanisms in *E. coli,* such as efflux pumps and mobile genetic elements and the need for resilience against the emergence of unforeseen variants, a new approach utilizing whole-genome sequencing (WGS) is being adopted to characterize AMR genes, mobile elements, and pathogen diversity at the genomic level [11].

Infections caused by DEC are typically self-limiting and primarily managed with oral rehydration therapy (ORT) to prevent dehydration. Antibiotic therapy is generally contraindicated for infections caused by EHEC due to the risk of exacerbating the disease through Shiga toxin release, which can lead to severe complications such as haemolytic uremic syndrome (HUS) [12]. For other DEC pathotypes, antibiotic treatment may be considered for patients with severe or persistent infections. However, the increasing prevalence of antibiotic resistance among *E. coli* strains globally may limit the effectiveness of this approach. The extensive use of antibiotics has contributed to the emergence and dissemination of multidrug-resistant *E. coli*, particularly strains resistant to fluoroquinolones and those producing extended-spectrum β-lactamases (ESBL) or carbapenemases, which represent a significant global health threat [3,9].

Bhutan, despite its abundant fresh water resources, faces persistent challenges in ensuring safe drinking water, particularly in rural and peri-urban areas. Recent national surveillance data indicate that only 52.8% of urban and rural water samples meet microbial standards, with *E. coli* contamination prevalent, especially during the monsoon season [13]. This highlights vulnerabilities in water treatment and distribution systems, and these environmental reservoirs may serve as critical sources for DEC transmission, facilitating the spread of both pathogenic and resistant strains. The role of animals and food products in the transmission of DEC is well established, with studies documenting the presence of MDR *E. coli* in livestock, poultry, raw milk, and food processing environments [14,15]. In Bhutan, the increasing demand for livestock products and cross-border trade with India have heightened the risk of zoonotic transmission and the introduction of high-risk clones. Surveillance data from neighbouring countries indicate that animal-derived foods, particularly raw milk and eggs, are frequently contaminated with EPEC, ETEC, and EAEC, often harbouring both resistance and virulence genes [15,16].

The rise in AMR is a growing concern and highlights the urgent need for continuous surveillance and research. Previous research indicates significant variations in antibiotic resistance profiles across different regions, with many developing countries reporting higher resistance levels. Surveillance data from 2016 to 2022 showed DEC and Rotavirus as the leading causes of diarrhea, with significant resistance patterns observed in amoxicillin, ampicillin and nalidixic acid. However, these isolates were not characterized and lacked genotypic characterization [17]. Bhutan has adopted the One Health approach to effectively combat AMR, which includes collaborative surveillance programmes, integrated AMR action plans, and public awareness campaigns to promote responsible antibiotic use and enhance infection control measures [18]. Despite these initiatives, there are multiple reports indicating inappropriate use of antibiotics and evidence of highly prevalent AMR pathogens in clinical settings. A study on AMR of uro-pathogens in the Jigme Dorji Wangchuck National Referral Hospital (JDWNRH) showed resistance of *E. coli* to at least two commonly used antibiotics in 80% of the samples [19]. Similarly, a report from the animal sector showed two ESBL (CTX-M-15)-producing *E. coli* strains isolated amongst 83 fecal samples from breeding pigs in Bhutan [20]. More recently, the Capturing Data on Antimicrobial Resistance Patterns and Trends in Use in Regions of Asia (CAPTURA) country report showed that the resistance rate of *E. coli* to third-generation cephalosporins was 66% in 2022 [21]. Despite multiple reports of AMR, there exists a notable gap in comprehensive genomic studies on these pathogens in Bhutan.

Understanding the genomic features and AMR profiles of DEC in Bhutan is crucial for guiding evidence-based interventions and combating AMR effectively. WGS offers a robust approach to elucidate the genomic anatomy of bacterial pathogens. We aim to identify virulence factors and resistomes that are critical for guiding empirical therapy and AMR surveillance efforts in Bhutan.

## 2. Materials and Methods

### 2.1. Source of Isolates

**Figure 1:**
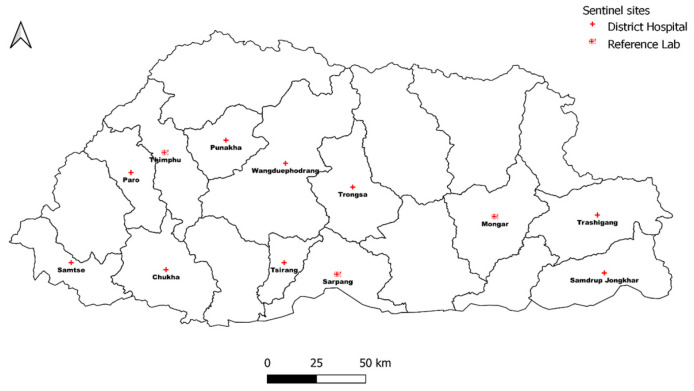
A district map of Bhutan showing National Diarrheal Disease Surveillance Sites. Referral hospitals and district hospitals participating are indicated on the map.

DEC strains were isolated from fecal samples collected from patients visiting the national diarrheal disease surveillance (NDDS) sentinel hospitals presenting with three or more episodes of loose or liquid stools within a 24 h period from January to December 2023. The NDDS is an ongoing activity implemented as part of a public health intervention, covering 12 designated sentinel hospitals (Figure 1).

### 2.2. Phenotypic Characterization and Antimicrobial Susceptibility Testing (AST)

All fecal samples were tested for a panel of eight enteric bacterial pathogens, including *Shigella* spp., *Salmonella* spp., *Campylobacter* spp., *Yersinia enterocolitica*, *Aeromonas* spp., *Enterococcus* spp., *Plesiomonas* spp., and *E. coli*, using conventional culture-based microbiological methods. Appropriate selective and differential media were used for each target organism, followed by incubation under organism-specific conditions. Presumptive isolates were identified based on colony morphology, Gram staining, and standard biochemical tests following the procedures described in the Clinical and Laboratory Standards Institute (CLSI) manual and standard clinical microbiology laboratory protocols (https://www.who.int/publications/i/item/WHO-CDS-CSR-RMD-2003.6, accessed on 15 January 2026). Identification of DEC was done using a multiplex PCR assay (primer sequences provided in Table S1) on the isolates that were confirmed to have *E. coli* using the aforementioned method. In total, 29 DEC were isolated in 2023 and included in this study. For phenotypic AST, the isolates were tested against ten antibiotics from eight different classes, using the modified Kirby–Bauer disc diffusion method on Mueller–Hinton agar following Clinical and Laboratory Standards Institute (CLSI) guidelines. The antibiotics tested included ampicillin (10 µg), cefazolin (30 μg), cephalexin (30 μg), gentamicin (10 μg), ciprofloxacin (5 µg), ceftriaxone (30 μg), nalidixic acid (30 μg), and trimethoprim–sulfamethoxazole (25 μg). The results obtained were classified as resistant, intermediate, or susceptible based on CLSI standard reference values and the *E. coli* strain, with ATCC 25922 used as a control.

### 2.3. Next-Generation Sequencing and Bioinformatics Analysis

Isolates were stored at the Royal Centre for Disease Control (RCDC) biorepository at −80 °C until processing. For WGS, isolates were revived and cultured on MacConkey agar, after which single colonies were re-streaked onto fresh plates to ensure purity. A purified colony was then inoculated into Luria–Bertani (LB) broth and incubated at 37 °C overnight. Genomic DNA was extracted from overnight cultures using the QIAamp DNA Mini Kit (QIAGEN, Hilden, Germany) according to the manufacturer’s instructions. DNA concentration and purity were assessed by Qubit fluorometer. Paired-end libraries were prepared using Illumina DNA prep and sequenced on an Illumina MiSeq using V2 chemistry [22]. Library fragment sizes were checked prior to sequencing using TapeStation 4150 (Agilent Technologies, Santa Clara, CA, USA). Raw reads were inspected and trimmed using fastp v1.3.3 [23] with default settings to remove adapters and low-quality bases. Read quality metrics, total read counts, and estimated genome coverage were recorded for each isolate using seqkit v2.1.0 [24]. Assemblies were considered suitable for downstream analysis if they met the following criteria: (i) a total genome size within the expected species range (4.5–5.5 Mb), (ii) an N50 value greater than 100 kb, and (iii) sufficient resolution to confidently resolve core-genome multilocus sequence types (cgMLST)/target marker genes. All downstream analyses were performed using Bohra (https://github.com/MDU-PHL/bohra, accessed on 10 October 2025), an ISO-certified microbial genomics pipeline written in Nextflow. Bohra was provided with a tab-delimited sample sheet containing isolate identifiers and paths to READ1 and READ2 (Illumina paired reads). The pipeline was executed with default settings unless stated otherwise. Key tools and versions used included shovill 1.1.0 (assembly) [25,26] snippy v.4.4.5 (read mapping and variant calling) [27], snp-dists v.0.8.2 (pairwise SNP distances), KMC v.3.2.1 (k-mer counting) [28], Kraken2 v.2.1.2 (taxonomic identification) [29], Ectyper 1.0.0 database version 1.0 (Serotyping) (https://github.com/phac-nml/ecoli_serotyping, accessed on 10 October 2025), abriTAMR v.1.0.20 (https://github.com/MDU-PHL/abritamr, accessed on 10 October 2025) using AMRFinderPlus (virulence factor and AMR identification) (GitHub–MDU-PHL/abritamr: A pipeline for running AMRfinderPlus and collating results into functional classes GitHub) [30], and mlst v.2.23.0 (https://github.com/tseemann/mlst, accessed on 10 October 2025) using the Achtman seven-gene *E. coli* typing scheme (https://enterobase.warwick.ac.uk, accessed on 10 October 2025) [30]. The pipeline generated a maximum-likelihood phylogeny using IQ-TREE multicore v.2.1.4 [31] and the *E. coli* strain K-12 (NC_000913.3) as the reference at default settings with the General Time Reversible (GTR) model and 1000 bootstrap replicates. The tree produced from Bohra was finally annotated using R v4.5.1 [32]. *E. coli* phylogroup assignment was determined using the ClermonTyper web interface hosted by CATIBioMed (IAME UMR 1137) and cross-checked against Bohra typing outputs (http://clermontyping.iame-research.center/, accessed on 10 October 2025) [33].

### 2.4. Sequence Data Submission

Raw sequence data in FASTQ format and associated de-identified metadata from isolates were submitted to the NCBI Sequence Read Archive (SRA) under BioProject PRJNA1302017 (SRA ID SRR34873955-SRR34873974), as shown in the Supplementary Table (Table S2).

## 3. Results

### 3.1. Distribution of DEC Pathotypes and Phenotypic AST

**Figure 2:**
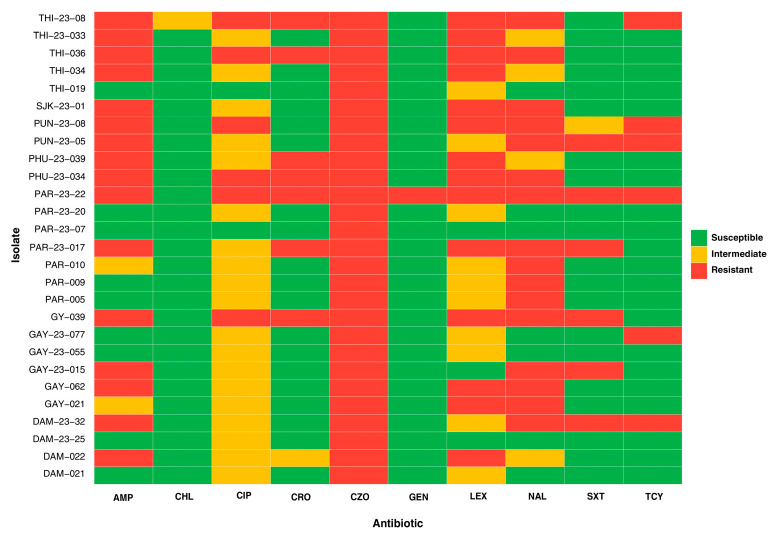
Phenotypic antibiogram of the DEC isolates. Phenotypes are shown as green for susceptible, yellow for intermediate and red for resistant. Antibiotics are indicated by a three-letter code: AMP, ampicillin; CZO, cefazolin; CRO, ceftriaxone; LEX, cephalexin; CHL, chloramphenicol; CIP, ciprofloxacin; GEN, gentamicin; NAL, nalidixic acid; TCY, tetracycline; SXT, trimethoprim–sulfamethoxazole.

From the 347 samples collected from diarrheal patients during the study period for the National Diarrheal Disease Surveillance, 29 were identified as DEC pathotypes through PCR. The most prevalent pathotypes were EPEC (37.0%) and EAEC (33.3%), followed by EHEC (14.8%) and EIEC (11.1%), with ETEC being the least frequent (3.7%). Phenotypic AST revealed widespread resistance to multiple commonly used antibiotics. All isolates exhibited resistance to cefazolin (CZO). Resistance to ampicillin (AMP), ceftriaxone (CRO), cephalexin (LEX), chloramphenicol (CHL), ciprofloxacin (CIP), gentamicin (GEN), nalidixic acid (NAL), tetracycline (TCY), and trimethoprim–sulfamethoxazole (SXT) varied among isolates and pathotypes. Notably, EAEC and EPEC isolates consistently displayed resistance to β-lactams, while susceptibility to ciprofloxacin, tetracycline, and trimethoprim–sulfamethoxazole was variable. Some isolates showed multidrug resistance patterns, including resistance to three or more antibiotic classes but most remained susceptible to GEN (Figure 2).

### 3.2. Whole-Genome Sequencing (WGS) of DEC Isolates

All 29 DEC isolates were sequenced using Illumina MiSeq and processed using the Bohra pipeline. The *Morganella morganii* and *Aeromonas* spp. were identified from the WGS of two isolates and hence removed from the downstream analysis. Raw sequencing output ranged from 0.55 M to 2.26 M reads per isolate, with mean read lengths of 146–236 bp and high-quality scores with >85–95% of bases above Q30. The average estimated sequencing depth was 52.3×. Genome assemblies ranged between 4.68 Mbp and 5.44 Mbp, with contig counts from 110 to 400, with the exception of one isolate, which was highly fragmented with 896 contigs. N50 values ranged from 68,402 bp to 164,412 bp. The detailed assembly quality statistics of DEC isolates are shown in Table S3.

### 3.3. Phylogenetic Groups, Serotype, and MLST of DEC Isolates

MLST revealed considerable genetic diversity within the collection, with 24 different sequence types, including ST10, ST21, and ST1312, and two isolates showed the same ST1125 (Table 1).

**Figure 3:**
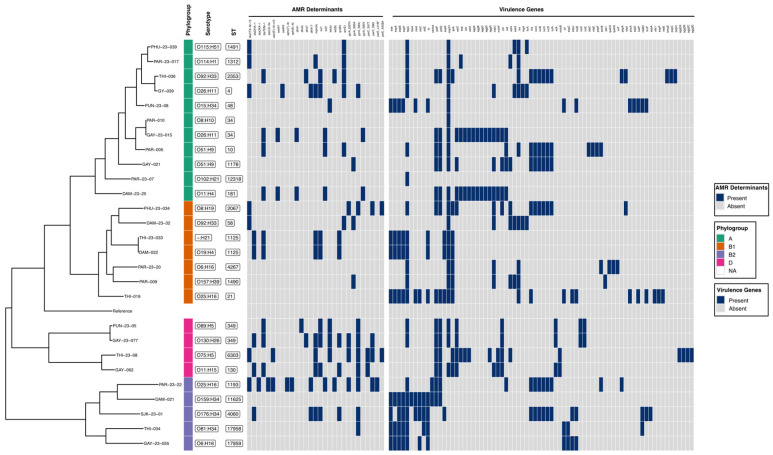
Maximum-likelihood tree of DEC isolates based on core-genome SNPs, mid-point-rooted with *E. coli* MG1655 (K 12) as the reference, and generated within the Bohra pipeline using IQ-TREE with the GTR model and 1000 ultrafast bootstrap replicates. Isolates are annotated by phylogroup (coloured highlights), sequence type (ST), serotype (labels next to ST) and resistance genes.

Two isolates were non-typeable using the Achtman scheme and were assigned novel sequence types ST17958 and ST17959 (*a novel ST identified in this study) via Enterobase submission. WGS further identified 22 distinct serotypes across the sequenced isolates. Phylogenetic analysis of the core genome SNPs classified the isolates into four major phylogroups, with the majority of isolates falling within phylogroup A (*n* = 11, 40.7%), followed by B1 (*n* = 7, 25.9%), B2 (*n* = 5, 18.5%) and D (*n* = 4, 14.8%) (Figure 3).

### 3.4. Antimicrobial Resistance Genes and Virulence Factors

All isolates (*n* = 27) were analyzed for AMR genes and showed a diverse range of ARGs in multiple antimicrobial classes (Table 2). Aminoglycoside resistance genes were detected in several isolates, including *aac*(6′)*-Ib-cr5*, *aac*(3)*-IIe*, *aadA*, *aadA5*, *aph*(3′)*-Ib*, and *aph*(6)*-Id*, conferring predicted resistance to streptomycin, gentamicin, tobramycin, and other aminoglycosides. Resistance to β-lactam antibiotics was widely spread. The narrow-spectrum *bla*_EC_ (*n* = 11), *bla*_DHA-1_ (*n* = 5), and *bla*_TEM-1_ (*n* = 9) were also present, while the ampicillin-associated variants *bla*_EC-15_ and *bla*_EC-18_ were identified in 13 and three isolates, respectively. Extended-spectrum β-lactamase (ESBL)-associated ARG *bla*_CTX-M-15_ was identified in 25.9% (*n* = 7) of the isolates, conferring resistance to third-generation cephalosporins and aztreonam. Among folate pathway antagonist ARGs, *sul1* (*n* = 8) and *sul2* (*n* = 4) were common, along with several trimethoprim resistance-associated *dfrA* gene variants, including *dfrA17* (*n* = 2), *dfrA1* (*n* = 2), *dfrA5* (*n* = 1), and *dfrA7* (*n* = 2). The macrolide resistance gene *mph*(A) was detected in eight isolates. Genes associated with quinolone resistance were also present. The plasmid-mediated genes *aac*(6′)*-Ib-cr5* (*n* = 2) and *qnrS1* (*n* = 6) were identified, while chromosomal mutations in *gyrA* were the most common quinolone resistance determinant, detected in 14 isolates. For tetracycline resistance, both *tet*(A) (*n* = 4) and *tet*(B) (*n* = 2) were present in the isolates analyzed. Several ARGs associated with other antimicrobial classes or multidrug resistance mechanisms were also identified. These included *marR_*S3N (*n* = 3), associated with multiple agents, including ampicillin and chloramphenicol, as well as Fosfomycin resistance-related mutations such as *glpT_*E448K (*n* = 18) and *uhpT_*E350Q (*n* = 5). The *pmrB* gene, associated with colistin resistance, was found in 12 isolates. Chloramphenicol resistance genes *catB3* and *catA1* were each identified in one isolate. Notably, efflux pump-related genes were highly prevalent, with *arcF* detected in 26 isolates, *mdtM* in 22 isolates, and *emrD* in nine isolates. No carbapenem resistance genes were detected in this study.

**Table 2:** Frequency of antimicrobial resistance (AMR) genes.

Antimicrobial Class	ARG	Phenotype (Agent)	Frequency (*n* = 27)
Aminoglycoside	*aac*(6′)*-Ib-cr5*	Streptomycin, Amikacin, Netilmicin, Sisomicin, Dibekacin	2
	*aac*(3)*-IIe*	Gentamicin, Tobramycin	1
	*aadA1*	Streptomycin	2
	*aadA5*	Streptomycin	1
	*aph*(3′)*-Ib*	Streptomycin	1
	*aph*(6)*-Id*	Streptomycin, Tobramycin	1
Beta-lactam (β-lactam)	*bla*_EC_	Narrow-spectrum	11
	*bla*_EC-15_	Ampicillin	13
	*bla*_EC-18_	Ampicillin	3
	*bla*_DHA-1_	Ampicillin	5
	*bla*_TEM-1_	Amoxicillin, Ampicillin, Piperacillin, Ticarcillin, Cephalothin	9
	*bla*_OXA-1_	Amoxicillin, Amoxicillin + Clavulanic acid, Ampicillin, Ampicillin + Clavulanic acid, Cefepime, Piperacillin, Piperacillin + Tazobactam	1
	*bla*_CTX-M-15_	Amoxicillin, Ampicillin, Cefepime, Cefotaxime, Ceftazidime, Piperacillin, Aztreonam, Ticarcillin, Ceftriaxone	7
Folate pathway antagonist	*sul1*	Sulfamethoxazole	8
	*sul2*	Sulfamethoxazole	4
	*dfrA17*	Trimethoprim	2
	*dfrA1*	Trimethoprim	2
	*dfrA5*	Trimethoprim	1
	*dfrA7*	Trimethoprim	2
Macrolide	*mph(A)*	Erythromycin, Azithromycin, Spiramycin, Telithromycin	8
Quinolone	*aac*(6′)*-Ib-cr5*	Fluoroquinolone, Ciprofloxacin	2
	*qnrS1*	Ciprofloxacin	6
	*gyrA*	Ciprofloxacin	14
Tetracycline	*tet*(A)	Tetracycline, Doxycycline	4
	*tet*(B)	Tetracycline, Doxycycline, Minocycline	2
Others	*marR_*S3N	Ampicillin, Chloramphenicol, Quinolone, Rifampin, Tetracycline	3
	*cyaA_*S352T	Fosmidomycin	2
	*glpT_*E448K	Fosfomycin	18
	*pmrB*	Cholistin	12
	*catB3*	Chloramphenicol	1
	*catA1*	Chloramphenicol	1
	*uhpT_*E350Q	Fosfomycin	5
	*arcF*	Efflux	26
	*emrD*	Efflux	9
	*mdtM*	Efflux	22

The table shows the frequency (*n*, count) of DEC isolates carrying specific AMR genes. Genes are grouped by major antimicrobial class, and frequencies were calculated as the number of isolates carrying each gene.

### 3.5. Other Virulence Factors

All isolates carried at least one acid resistance gene (*ymgB*), and multiple isolates harboured biocide resistance genes, including *emrE*, *qacE*, and *qacEdelta1*. Heat resistance genes were generally absent, except for specific stress-associated genes (*hdeD-GI*, *hsp20*, *kefB-GI*, etc.) in a few isolates. Metal resistance genes were detected variably, with copper (*pcoA–pcoE*, *pcoR–pcoS*) and silver (*silA–silS*) operons present in multiple isolates, suggesting potential adaptation to heavy-metal environments. Virulence genes were widespread, with notable prevalence of toxins (*ehxA*, *sat*, *vactox*), iron acquisition systems (*iucA-iucD*, *iutA*, *iroD*, *iroE*), and type III secretion system components (*espA-espF*, *espX1*, *nleA–nleC*, *tir*). The distribution of virulence determinants is shown in Table 3.

The table summarizes virulence genes identified across all isolates, grouped according to the number of isolates in which each gene was detected. Genes listed together share the same detection frequency. Frequencies represent the total number of isolates possessing each gene, classified through WGS.

## 4. Discussion

This study presents the first comprehensive WGS-based characterization of DEC isolates from Bhutan, providing detailed insights into the genomic diversity, pathotype distribution, and phylogenetic structure of DEC. Globally, DEC is classified into seven major pathotypes: ETEC, EPEC, EAEC, EHEC, EIEC, DAEC and AIEC. The relative prevalence of these pathotypes varies by region, age group, and environmental context [8]. In Asia, ETEC, EPEC, and EAEC are consistently reported as the predominant causes of childhood diarrhea, with ETEC and EAEC particularly associated with high morbidity and mortality in low- and middle-income countries [34,35,36,37]. The DEC isolates in this study exhibited a distribution of pathotypes similar to regional trends. *E. coli* is structured into at least eight phylogroups (A, B1, B2, C, D, E, F, G), with phylogroups A, B1, and D most commonly associated with intestinal pathogenic strains, while B2 and D are often linked to extraintestinal pathogenic *E. coli* (ExPEC) [38]. In the Bhutanese isolates, phylogroups A and B1 were predominant, consistent with the reported literature elsewhere [39,40]. This predominance suggests a reservoir of lineages capable of thriving in both human hosts and environmental niches, potentially facilitating transmission through contaminated water and food.

Sequence typing, based on MLST, showed diverse STs among Bhutanese DEC, such as ST10, ST38, and ST69, which are frequently implicated in both intestinal and extraintestinal infections [41,42]. This diversity underscores the dynamic nature of DEC populations in Bhutan and the potential for both local evolution and introduction of international clones.

The high presence of strains resistant to multiple antimicrobial agents and ESBL production among DEC is a critical public health threat, complicating empirical treatment and increasing the risk of adverse clinical outcomes. The detection of *bla*_CTX-M-15_ as the dominant ESBL gene in our study is consistent with its global emergence as the most prevalent CTX-M variant [42,43,44]. CTX-M-15 has been widely reported in Asia, Africa, Europe, and the Americas, often associated with pandemic clones such as ST131 and ST10. In Asia, the prevalence of CTX-M-producing *E. coli* was estimated at around 56%, with rates as high as 94% in Iran and 60% in China [43]. Many studies highlighted the association of *bla*_CTX-M-15_ in Enterobacteriaceae with mobile genetic elements such as insertion sequences (*ISEcp1*, IS26), integrons (class 1), and *IncF* family plasmids, which facilitate horizontal gene transfer (HGT) and rapid dissemination [45,46,47]. The occurrence of *bla*_CTX-M-15_ with other resistance determinants such as aminoglycoside-modifying enzymes, sulfonamide resistance genes (*sul1*, *sul2*), and plasmid-mediated quinolone resistance (*qnrS1*) has been documented in both clinical and environmental isolates, further complicating the challenge of resistance to multiple antimicrobial agents [42,46,48,49].

Fluoroquinolone resistance in *E. coli* is primarily mediated by chromosomal mutations in the quinolone resistance-determining regions (QRDRs) of *gyrA* and *parC*, with S83L and D87N in *GyrA* and S80I in *ParC* being the most prevalent mutations [50,51]. These mutations confer high-level resistance to ciprofloxacin and other fluoroquinolones, and their co-occurrence is often required for clinically significant resistance [50,51]. In our study, isolates exhibited both QRDR mutations and PMQR genes, reflecting the global trend of increasing fluoroquinolone resistance among Enterobacteriaceae. Regional studies report fluoroquinolone resistance rates of 34.5% to 80.9% among DECs in Asia, with similar patterns observed in Africa and South America [6,39,48]. The co-occurrence of ESBL and fluoroquinolone resistance is a major problem, as it limits the efficacy of both first- and second-line therapies for diarrheal disease [42,43].

The resistance to sulfonamides and trimethoprim is mediated by the acquisition of *sul1*, *sul2*, and *dfrA* genes [42,48]. These genes are highly prevalent among DEC isolates in Asia and Africa, with studies reporting detection rates exceeding 50% in both clinical and foodborne isolates [44,48,52]. Our isolates harboured both *sul1* and *sul2*, as well as *dfrA1* and *dfrA10*, conferring high-level resistance to cotrimoxazole (trimethoprim–sulfamethoxazole), a commonly used empirical therapy for diarrheal disease in the country [53].

The pathogenicity of DEC is determined by a complex interplay of virulence factors that mediate adherence, invasion, toxin production, iron acquisition, and immune evasion [42,43]. Our isolates were found to possess a diverse array of virulence genes, including *esp*, *tir*, *iha*, *aap*, and multiple iron acquisition systems, reflecting both classical and emerging mechanisms of pathogenesis. The *esp* (*E. coli* secreted protein) and *tir* (translocated intimin receptor) genes are central to the attaching and effacing (A/E) phenotype characteristics of EPEC and EHEC, mediating intimate adherence to intestinal epithelial cells and the formation of pedestal-like lesions [10]. The *eae* gene encodes intimin, an outer membrane adhesin essential for A/E lesion formation, while *bfp* (bundle-forming pilus) facilitates initial adherence in typical EPEC [8,9]. The detection of these genes in our isolates confirms the presence of classical EPEC and EHEC pathotypes, with the potential to cause severe and persistent diarrhea, particularly in young children [37]. The most commonly detected virulence genes from DEC isolates in our study include *fdeC*, *espX1*, *ybtP*, *ybtQ* and *lpfA*, which correlate with the study of AMR genes from five countries by Cui K et al. [54].

### Limitations

This study has a few limitations. First, the sample size is relatively small (29 isolates), which may limit the generalizability of the findings and the detection of less prevalent pathotypes or resistance mechanisms. Second, the absence of parallel metagenomic sequencing limits the ability to capture the full spectrum of DEC diversity and the contribution of unculturable or minority populations. Additionally, few assemblies (e.g., THI-019 with 896 contigs) were highly fragmented due to short-read sequencing limitations in repetitive regions. However, our downstream analysis relied on gene screening/phylogenetics, which remains robust in fragmented drafts. Future long-read sequencing will help fully close these genomes. The lack of comprehensive environmental and animal sampling precludes a full assessment of transmission pathways and the role of non-human reservoirs. Lastly, while WGS provides unparalleled resolution, challenges remain in the interpretation of resistance and virulence gene expression, the functional impact of mutations, and the integration of genomic data into routine clinical and public health practice.

## 5. Conclusions

This study provides the first whole-genome characterization of DEC in Bhutan, showing substantial genomic diversity, including predominant phylogroups A and B1 with 22 distinct serotypes. The isolates exhibited resistance to multiple antimicrobial agents, including ESBL production (primarily *bla*_CTX-M-15_), fluoroquinolone resistance, and sulfonamide/trimethoprim resistance, alongside a broad range of virulence determinants mediating adherence, toxin production, and iron acquisition.

These findings indicate that DECs in Bhutan represent a significant public health concern, with environmental and foodborne reservoirs likely contributing to transmission, underscoring the need for integrated genomic surveillance through the One Health approach, water safety interventions, and antimicrobial stewardship. Furthermore, research studies are recommended to study the possible transfer of resistance genes through food and water to generate high-quality evidence for policymakers’ evidence-based decision-making.

## Figures and Tables

**Table 1:** Distribution of STs among Bhutanese DEC isolates.

Isolate	ST	Allele 1	Allele 2	Allele 3	Allele 4	Allele 5	Allele 6	Allele 7
PAR-005	10	*adk*(10)	*fumC*(11)	*gyrB*(4)	*icd*(8)	*mdh*(8)	*purA*(8)	*recA*(2)
THI-034	17958 *	*adk*(142)	*fumC*(43)	*gyrB*(10)	*icd*(15)	*mdh*(30)	*purA*(14)	*recA*(137)
PAR-23-017	1312	*adk*(6)	*fumC*(11)	*gyrB*(4)	*icd*(8)	*mdh*(8)	*purA*(78)	*recA*(2)
THI-019	21	*adk*(16)	*fumC*(4)	*gyrB*(12)	*icd*(16)	*mdh*(9)	*purA*(7)	*recA*(7)
DAM-022	1125	*adk*(6)	*fumC*(4)	*gyrB*(15)	*icd*(18)	*mdh*(24)	*purA*(26)	*recA*(7)
GAY-23-077	349	*adk*(34)	*fumC*(36)	*gyrB*(39)	*icd*(87)	*mdh*(67)	*purA*(16)	*recA*(4)
PAR-23-20	4267	*adk*(6)	*fumC*(6)	*gyrB*(4)	*icd*(16)	*mdh*(24)	*purA*(8)	*recA*(7)
GAY-23-055	17959 *	*adk*(15)	*fumC*(14)	*gyrB*(13)	*icd*(15)	*mdh*(18)	*purA*(14)	*recA*(13)
DAM-23-32	58	*adk*(6)	*fumC*(4)	*gyrB*(4)	*icd*(16)	*mdh*(24)	*purA*(8)	*recA*(14)
THI-036	2353	*adk*(6)	*fumC*(5)	*gyrB*(4)	*icd*(107)	*mdh*(8)	*purA*(8)	*recA*(2)
SJK-23-01	4060	*adk*(76)	*fumC*(24)	*gyrB*(244)	*icd*(89)	*mdh*(17)	*purA*(8)	*recA*(79)
DAM-021	11625	*adk*(129)	*fumC*(21)	*gyrB*(13)	*icd*(97)	*mdh*(17)	*purA*(14)	*recA*(896)
DAM-23-25	181	*adk*(8)	*fumC*(11)	*gyrB*(4)	*icd*(8)	*mdh*(7)	*purA*(8)	*recA*(6)
PAR-010	34	*adk*(10)	*fumC*(11)	*gyrB*(4)	*icd*(1)	*mdh*(8)	*purA*(8)	*recA*(2)
GY-039	4	*adk*(6)	*fumC*(5)	*gyrB*(4)	*icd*(8)	*mdh*(8)	*purA*(8)	*recA*(2)
PAR-23-07	12318	*adk*(879)	*fumC*(70)	*gyrB*(5)	*icd*(10)	*mdh*(8)	*purA*(1)	*recA*(2)
PUN-23-08	48	*adk*(6)	*fumC*(11)	*gyrB*(4)	*icd*(8)	*mdh*(8)	*purA*(8)	*recA*(2)
GAY-23-015	34	*adk*(10)	*fumC*(11)	*gyrB*(4)	*icd*(1)	*mdh*(8)	*purA*(8)	*recA*(2)
PHU-23-034	2067	*adk*(6)	*fumC*(95)	*gyrB*(3)	*icd*(18)	*mdh*(11)	*purA*(122)	*recA*(2)
PHU-23-039	1491	*adk*(6)	*fumC*(11)	*gyrB*(4)	*icd*(223)	*mdh*(8)	*purA*(78)	*recA*(2)
GAY-021	1178	*adk*(10)	*fumC*(27)	*gyrB*(5)	*icd*(8)	mdh(12)	purA(1)	recA(2)
PAR-23-22	1193	adk(14)	fumC(14)	gyrB(10)	*icd*(200)	*mdh*(17)	*purA*(7)	*recA*(10)
PUN-23-05	349	*adk*(34)	*fumC*(36)	*gyrB*(39)	*icd*(87)	*mdh*(67)	*purA*(16)	*recA*(4)
THI-23-08	6303	*adk*(18)	*fumC*(22)	*gyrB*(20)	*icd*(6)	*mdh*(5)	*purA*(354)	*recA*(4)
THI-23-033	1125	*adk*(6)	*fumC*(4)	*gyrB*(15)	*icd*(18)	*mdh*(24)	*purA*(26)	*recA*(7)
PAR-009	1490	*adk*(9)	*fumC*(6)	*gyrB*(204)	*icd*(131)	*mdh*(24)	*purA*(8)	*recA*(7)
GAY-062	130	*adk*(18)	*fumC*(22)	*gyrB*(20)	*icd*(6)	*mdh*(5)	*purA*(5)	*recA*(4)

* a novel ST identified in this study.

**Table 3:** Frequency of virulence-associated genes.

Category	Gene/Gene Cluster	Frequency (No. of Isolates, *n* = 27)
Acid tolerance	*ymgB*	20
Biocide resistance	*emrE*	13
	*qacE*	4
	*qacEΔ1*	6
Heat/stress response	*hdeD-GI*, *hsp20*, *kefB-GI*, *psi-GI*, *shsP*, *trxLHR*, *yfdX1*, *yfdX2*	1
Copper (pcoA–pcoS)	*pcoA*, *pcoB*, *pcoC*, *pcoD*, *pcoR*, *pcoS*	6
	*pcoE*	4
Silver (silA–silS)	*silA*, *silB*, *silE*, *silF*, *silP*, *silR*, *silS*	6
	*silC*	4
Mercury	*merC*, *merP*, *merR*, *merT*	5
Arsenic	arsD	2
Virulence factors	*fdeC*	26
	*espX1*	24
	*ybtP*, *ybtQ*	20
	*lpfA*	13
	*iha*, *iss*, *iucA*, *iucB*, *iucD*, *iutA*, *pic*	12
	*astA*, *tir*, *eae*	10
	*aaiC*, *espA*, *espB*, *espF*	9
	*capU*	8
	*mchF*	6
	*eatA*, *sat*, *toxB*, *espK*, *nleC*, *tccP*	5
	*espJ*, *nleA*, *aggR*, *aap*	4
	*nleB2*, *sslE*, *ltcA*, *mchB*, *aar*, *aatA*, *eilA*, *iroD*, *iroE*	3
	*senB*, *papA*, *espC*, *espI*, *ehxA*, *nleB*, *vactox*, *lngA*, *aggA*, *aggB*, *aggC*, *aggD*, *ipaH5*	2
	*papF*, *papC*, *papH*, *ibeA*, *espP*, *efa1*, *etpD*, *sta1*, *sepA*, *ipaH2*, *agg3A*, *agg3B*, *agg3C*, *agg3D*, *bmaE*, *nfaE*	1

## Data Availability

All the raw data supporting these reported results are available on request basis. However, the gene accession number for the sequence data are available in SRA gene bank-NCBI.

## Supplementary Materials

The following supporting information can be downloaded at: https://www.mdpi.com/article/10.3390/microorganisms14071541/s1, Table S1: DEC primer sequences; Table S2: NCBI accession numbers; Table S3: Assembly Statistics.

## Abbreviations

The following abbreviations are used in this manuscript:

DEC	Diarrheagenic *Escherichia coli*
AMR	Antimicrobial resistance
WGS	Whole-genome sequencing
PCR	Polymerase chain reaction
GEMS	Global Enteric Multicentre Study
VFs	Virulence factors
EPEC	Enteropathogenic *E. coli*
EHEC	Enterohemorrhagic *E. coli*
ETEC	Enterotoxigenic *E. coli*
EIEC	Enteroinvasive *E. coli*
EAEC	Enteroaggregative *E. coli*
DAEC	Diffusely adherent *E. coli*
AIEC	Adherent-invasive *E. coli*
AST	Antimicrobial susceptibility testing
ORT	Oral rehydration therapy
HUS	Haemolytic uremic syndrome
ESBL	Extended-spectrum β-lactamases
JDWNRH	Jigme Dorji Wangchuck National Referral Hospital
CAPTURA	Capturing Data on Antimicrobial Resistance Patterns and Trends in Use in Regions of Asia
NDDS	National diarrheal disease surveillance
CLSI	Clinical and Laboratory Standards Institute
ATCC	American Type Culture Collection
NGS	Next-generation sequencing
RCDC	Royal Centre for Disease Control
LB	Luria–Bertani
DNA	Deoxyribonucleic acid
SNP	Single-nucleotide polymorphism
NCBI	National Center for Biotechnology Information
SRA	Sequence Read Archive
REBH	Research and Ethics Board of Health
CZO	Cefazolin
AMP	Ampicillin
CRO	Ceftriaxone
LEX	Cephalexin
CHL	Chloramphenicol
CIP	Ciprofloxacin
GEN	Gentamicin
NAL	Nalidixic acid
TCY	Tetracycline
SXT	Trimethoprim–sulfamethoxazole
MLST	Multilocus sequence typing
ExPEC	Extraintestinal pathogenic *E. coli*
QRDR	Quinolone resistance-determining region
MDR	Multidrug resistance
